# Supplementary material for: IL‐7‐dependent compositional changes within the γδ T cell pool in lymph nodes during ageing lead to an unbalanced anti‐tumour response
Source: EMBO Rep. 2019 Jul 8;20(8):e47379. doi: 10.15252/embr.201847379 (PMC6680116; doi:10.15252/embr.201847379)
Supplement: Supplementary file 1 — Appendix [file EMBR-20-e47379-s001.pdf]

APPENDIX

IL-7-dependent compositional changes of the γδ T cell pool in lymph nodes during ageing lead to an unbalanced anti-tumour response

Hung-Chang Chen[1], Nils Eling[1,2,*], Celia Pilar Martinez-Jimenez[1,3,4,*], Louise McNeill O'Brien[1], Valentina Carbonaro[1], John C. Marioni[1,2,3], Duncan T. Odom[1,3,5], and Maike de la Roche[1,§]

[1]University of Cambridge, Cancer Research UK Cambridge Institute, Robinson Way, Cambridge CB2 0RE, UK
[2]European Molecular Biology Laboratory, European Bioinformatics Institute (EMBL-EBI), Wellcome Genome Campus, Hinxton, Cambridge CB10 1SD, UK
[3]Wellcome Sanger Institute, Wellcome Genome Campus, Hinxton, Cambridge CB10 1SA, UK
[4]Helmholtz Pioneer Campus, Helmholtz Zentrum München, D-85764 Neuherberg, Germany
[5]German Cancer Research Center (DKFZ), Division of Signaling and Functional Genomics, 69120 Heidelberg, Germany

*These authors contributed equally to this study.
§Corresponding author. Email: maike.delaroche@cruk.cam.ac.uk (M.d.I.R.)

TABLE OF CONTENT

Supplementary Figures

Appendix Figure S1. CD44 and CD45RB expression by γδ T cells in peripheral lymph nodes (pLNs) identifies IL-17-producing (γδ17) and IFN-γ-producing (γδ1) lineages........................1

Appendix Figure S2. Characterisation of the γδ T cell pool in pLNs from young and old mice of an independently maintained ageing colony..2

Appendix Figure S3. Characterisation of the γδ T cell pool in the mesenteric LNs (mLNs) of young and old mice..3

Appendix Figure S4. Characterisation of the splenic γδ T cell pool in young and old mice......4

Appendix Figure S5. Isolation of γδ1 and γδ17 T cells from different γδ T cell subsets..........5

Appendix Figure S6. Differentially expressed genes between γδ1 and γδ17 T cell subsets isolated from young and old mice..6

Appendix Figure S7. Quality controls of repertoire analysis..7

Appendix Figure S8. IL-7-dependent proliferation of γδ17 T cells in pLNs of mid-aged mice..8

Appendix Figure S9. IL-17 production by tumour-infiltrating T cells……………………………9

Appendix Figure S10. Expression of PD-1 and Tim-3 by γδ T cells in the pLNs of young and old mice under homeostatic condition……………………………………..…………………..10

Appendix Figure S11. Activation of γδ T cell subsets in LL2 tumours and tumour-draining LNs of young and old mice…………………………………………………………………….………11

Supplementary Tables

Appendix Table S1. Antibodies used in this study……………………………………………..12

Appendix Table S2. Gene nomenclatures of TCRδ variable segments………………………..14

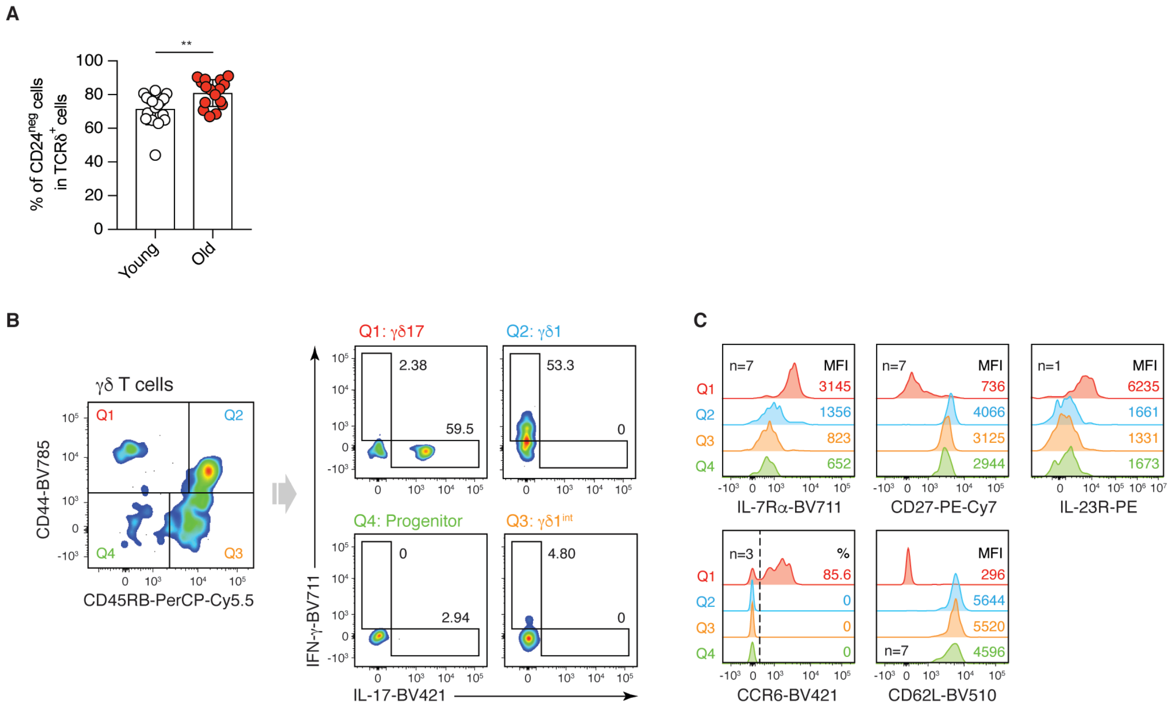

Appendix Figure S1. CD44 and CD45RB expression by γδ T cells in peripheral lymph nodes (pLNs) identifies IL-17-producing (γδ17) and IFN-γ-producing (γδ1) lineages. (A) Maturation status of γδ T cells in pLNs of young and old mice according to the expression of CD24. Results shown are collected from 8 independent experiments with 17 young and 16 old mice. Error bars represent SD. **(B)** γδ T cells were harvested from pLNs of young and old mice, stimulated with 50 ng/ml PMA and 1 μg/ml ionomycin in the presence of GolgiSTOP for 4 hours or left unstimulated. After 4 hours, cells were stained with live/dead and antibodies against cell surface markers followed by fixation, permeabilisation and staining for intracellular IL-17 and IFN-γ. FACS plots are representative of 6 independent experiments with 16 young mice. **(C)** Expression of characteristic lineage markers for γδ1 and γδ17 T cells by populations separated by CD44 and CD45RB expression. FACS plots shown are representative for the number of mice indicated.

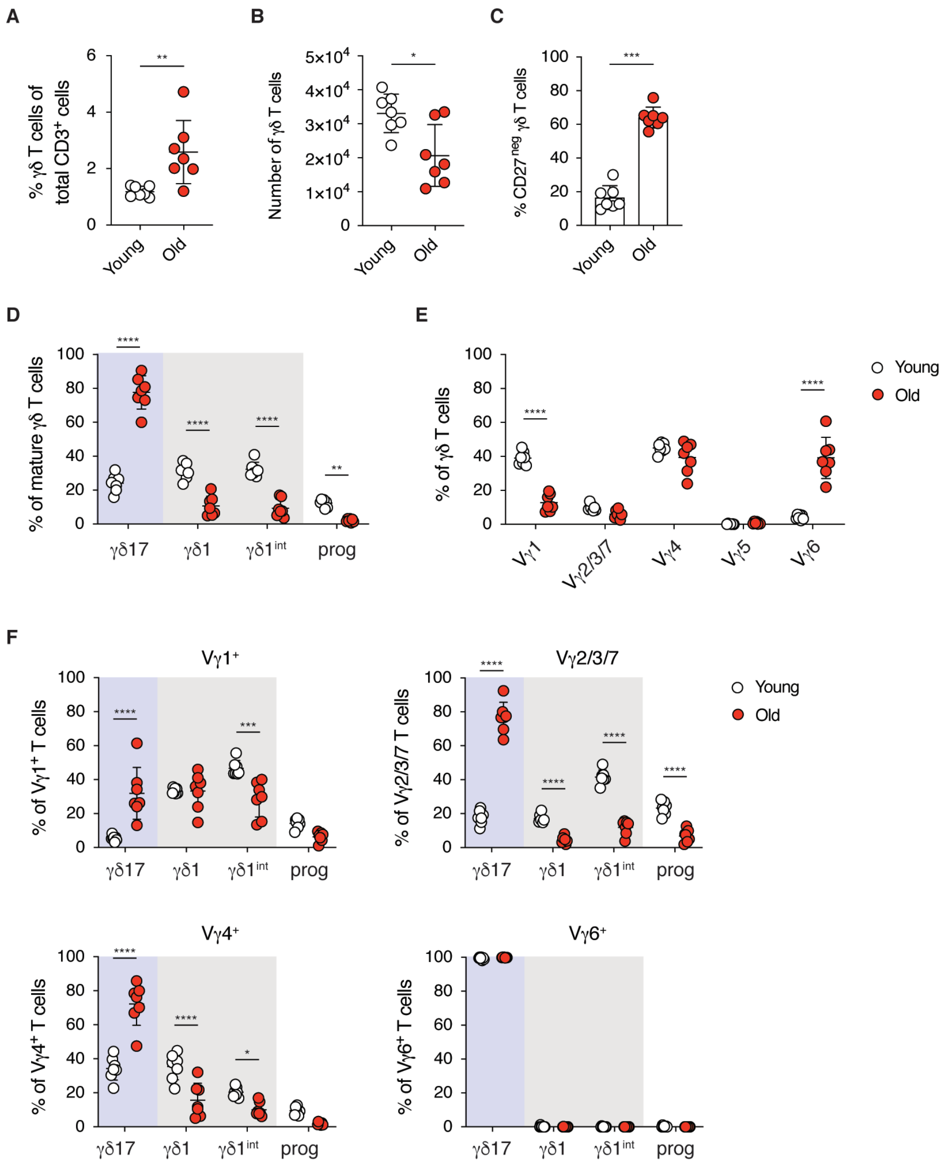

Appendix Figure S2. Characterisation of the γδ T cell pool in pLNs from young and old mice of an independently maintained ageing colony. (A) Proportion of γδ T cells in total mature CD3+ T lymphocytes in the pLNs of young and old mice. **(B)** Absolute numbers of γδ T cells in the pLNs of young and old mice. **(C and D)** γδ1 and γδ17 lineage commitment of mature γδ T cells in the pLNs of young and old mice. **(E)** Proportion of different γδ T cell subsets in total mature γδ T cells in the pLNs of young and old mice. **(F)** γδ1 and γδ17 lineage commitment of various γδ T cell subsets in the pLNs of young and old mice. Statistical significance for changes were assessed by Mann-Whitney test (A-C) or two-way ANOVA (D-F). Error bars represent SD. **p*<0.05; ***p*<0.01; ****p*<0.001; *****p*<0.0001

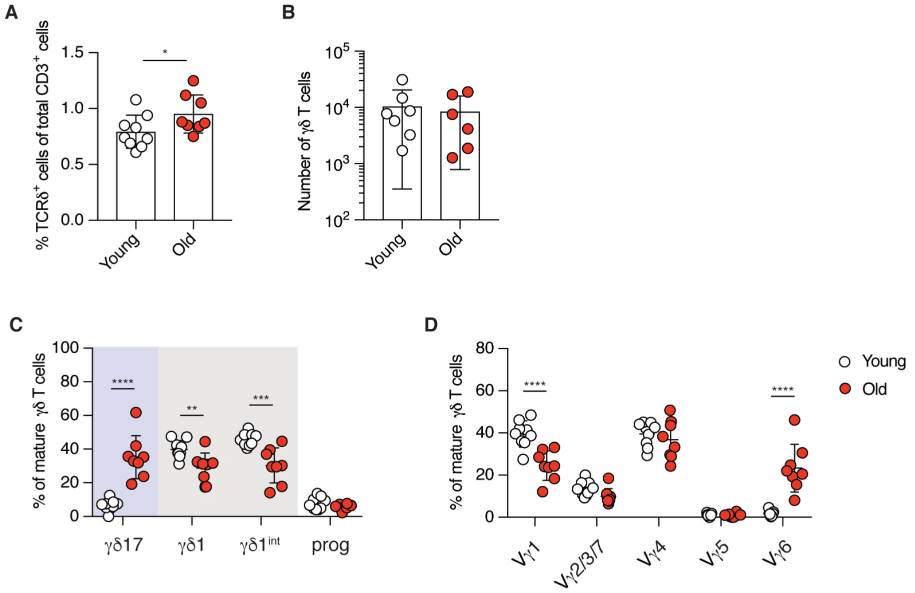

Appendix Figure S3. Characterisation of the γδ T cell pool in the mesenteric LNs (mLNs) of young and old mice. (A) Proportion of γδ T cells in total CD3+ T lymphocytes in the mLNs of young and old mice. **(B)** Absolute numbers of γδ T cells in the mLNs of young and old mice. Results shown are collected from 4 independent experiments with 7 young and 6 old mice. **(C and D)** γδ1 and γδ17 lineage commitment (C) and proportion of γδ T cell subset (D) of mature γδ T cells in the mLNs of young and old mice. Results shown (C and D) are collected from 5 independent experiments with 9 young and 8 old mice. Statistical significance for changes were assessed by Mann-Whitney test (A and B) or two-way ANOVA (C and D). Error bars represent SD. *$p<0.05$; **$p<0.01$; ***$p<0.001$; ****$p<0.0001$

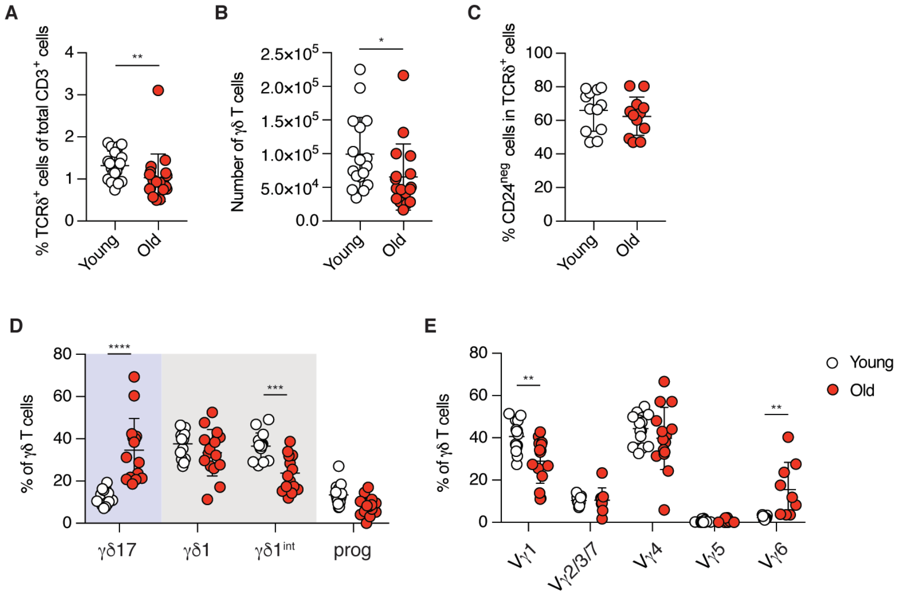

Appendix Figure S4. Characterisation of the splenic γδ T cell pool in young and old mice. (A) Proportion of γδ T cells in total CD3+ T lymphocytes in the spleen of young and old mice. **(B)** Absolute numbers of γδ T cells in the spleen of young and old mice. **(C)** Maturation status of γδ T cells in the spleen of young and old mice according to the expression of CD24. **(D)** γδ1 and γδ17 lineage commitment and **(E)** proportion of γδ T cell subset of mature γδ T cells in the spleen of young and old mice. Results shown are collected from 7 independent experiments with 17 young and 17 old mice. Statistical significance for changes were assessed by Mann-Whitney test (A-C) or two-way ANOVA (D and E). Error bars represent SD. *p<0.05; **p<0.01; ***p<0.001; ****p<0.0001

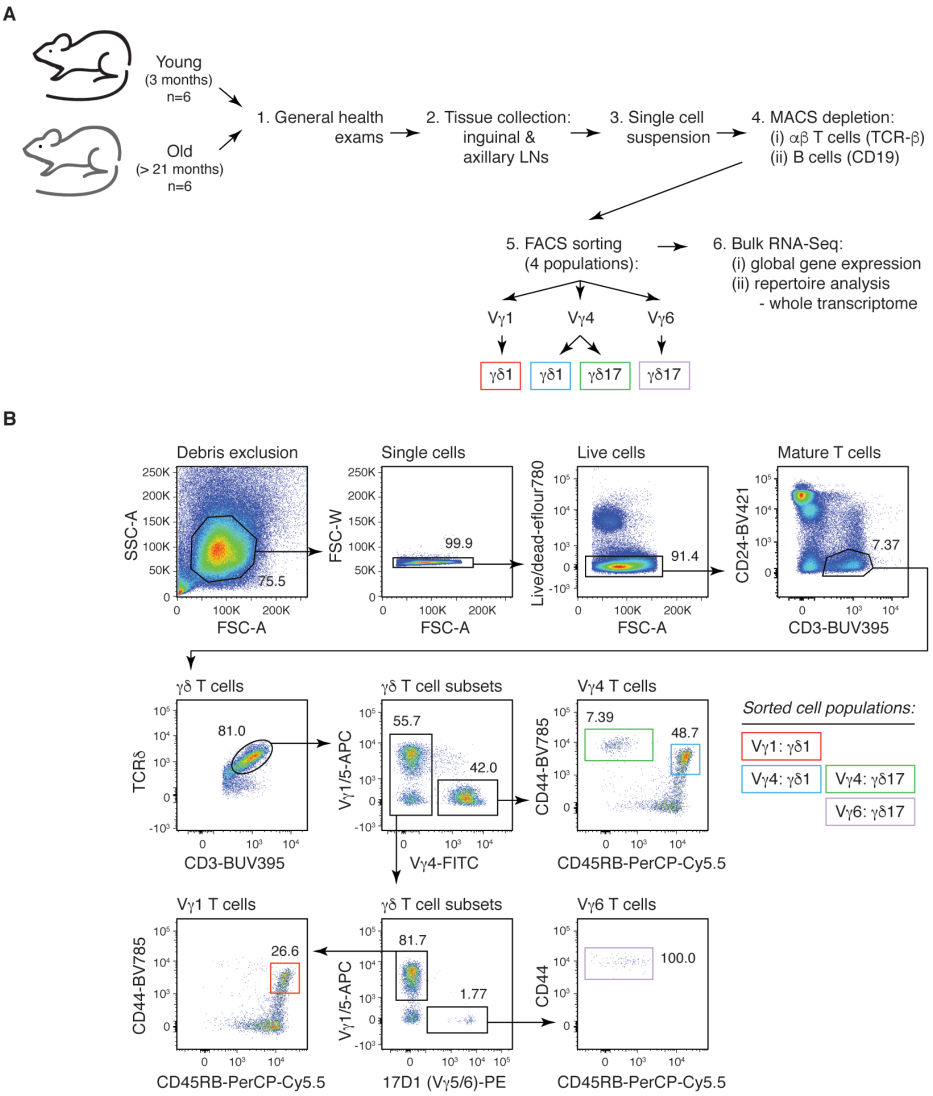

Appendix Figure S5. Isolation of γδ1 and γδ17 T cells from different γδ T cell subsets. (A) Four populations of γδ T cells were isolated from the pLNs of young and old mice for bulk RNA-Seq. **(B)** FACS gating strategy: Lymphocytes were gated by forward scatter (FSC-A) and side scatter (SSC-A). Cell doublets were excluded according to area and width of the forward scatter (FSC-A/FSC-W). Dead cells were removed using viability dye. From mature T lymphocytes (CD3^{+} CD24^{neg}), γδ T cells were determined by TCRδ expression. γδ T cells were then further segregated into 4 cell subsets according to their expression of different TCRγ chains. Vγ1^{+}, Vγ4^{+} and Vγ6^{+} T cells were separated by the staining profile of cells with antibodies against Vγ1^{+}, Vγ4^{+} and Vγ5^{+} TCR and 17D1 hybridoma supernatant against Vγ5/Vγ6 TCR. γδ1 (CD44^{+} CD45RB^{+}) and γδ17 (CD44^{hi} CD45RB^{neg}) T cells were characterised within Vγ1^{+}, Vγ4^{+} and Vγ6^{+} T cells. γδ1 T cells were isolated from Vγ1^{+} (red) and Vγ4^{+} (blue) T cell subsets and γδ17 T cells were isolated from Vγ4^{+} (green) and Vγ6^{+} (purple) T cell subsets.

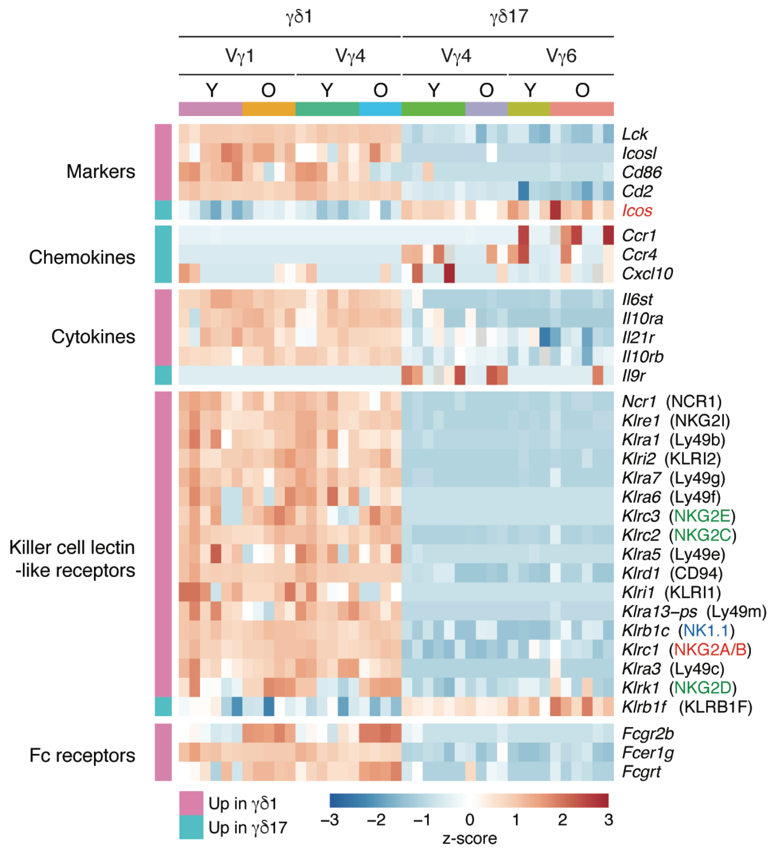

Appendix Figure S6. Differentially expressed genes between γδ1 and γδ17 T cell subsets isolated from young and old mice. Genes expressed at different levels in γδ1 and γδ17 T cells were identified by RNA-Seq analysis. Heatmap shows the Z-score scaled, normalised expression of selected marker genes.

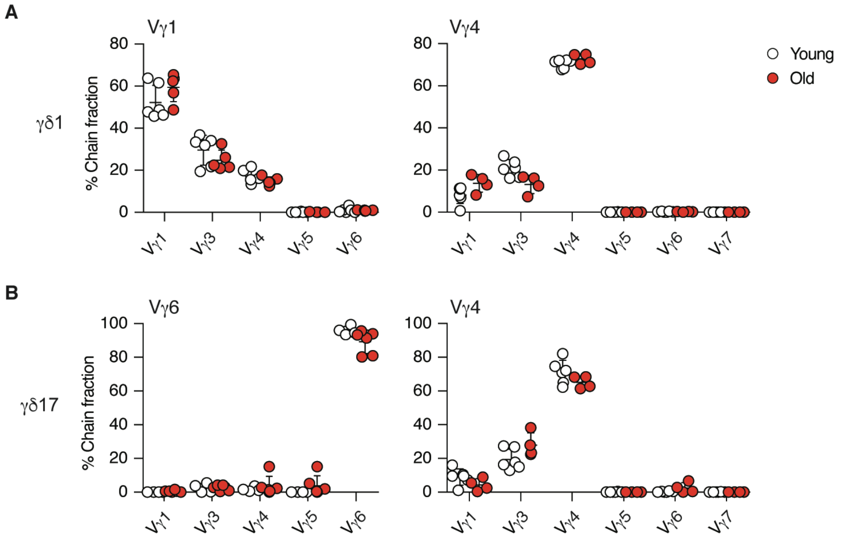

Appendix Figure S7. Quality controls of repertoire analysis. TCRγ and TCRδ chains were assembled from bulk RNA-Seq data of highly pure, FACS-sorted **(A)** Vγ1⁺ and Vγ4⁺ γδ1 T cells and **(B)** Vγ6⁺ and Vγ4⁺ γδ17 T cells. MiXCR was used in RNA-Seq mode to reconstruct TCRγ and TCRδ chains. As a quality control, the fraction of assembled TCRγ chains was plotted for each sample. Only T cell subset-specific sequences were used for further analysis. The detected non-specific sequences are likely due to alignment errors resulting from high level of homology between Vγ1 and Vγ3 (A). Error bars represent SD.

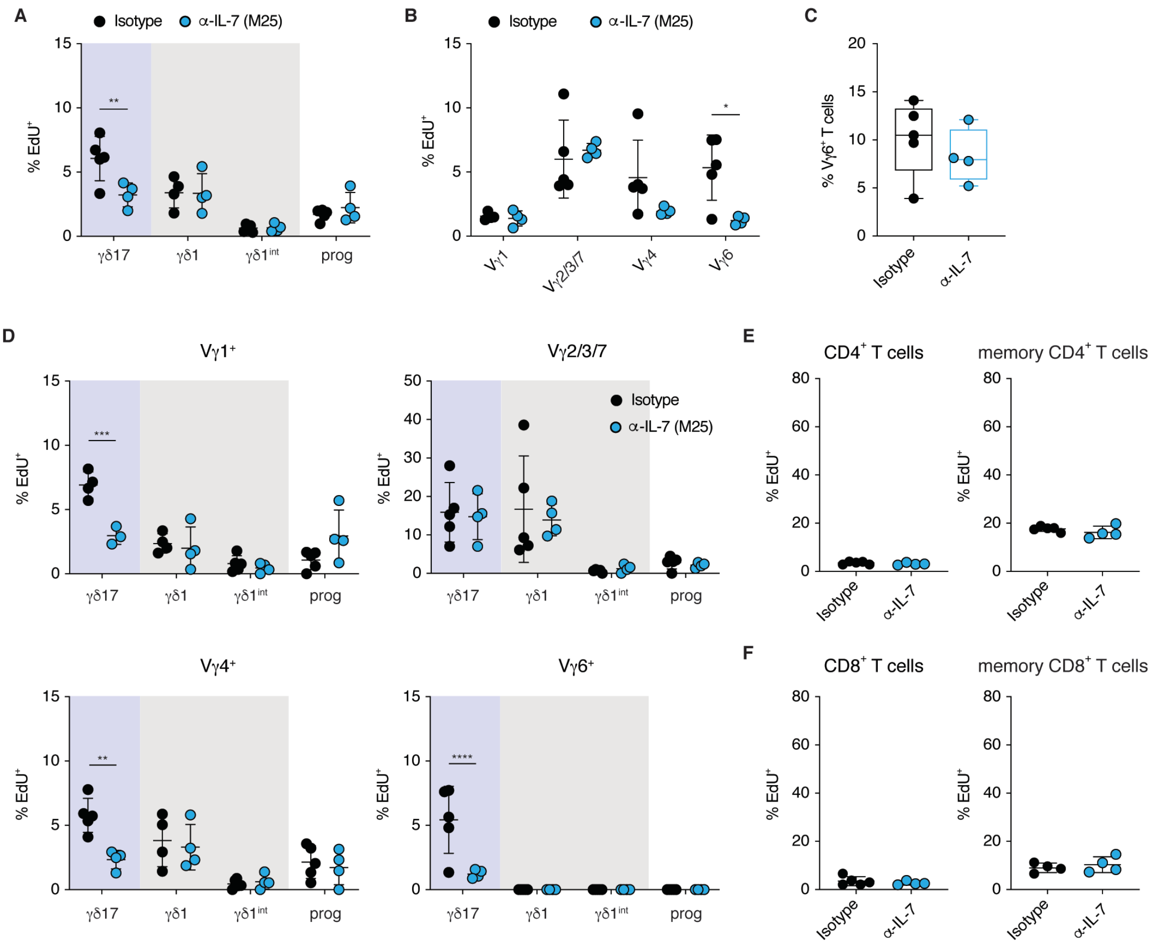

Appendix Figure S8. IL-7-dependent proliferation of γδ17 T cells in pLNs of mid-aged mice. Mid-aged mice (12 months old) were treated with control isotype IgG2b or with anti-IL-7 neutralising antibody, followed by EdU labelling over 3 days as described in Fig. 5I. **(A and B)** Proliferation of γδ1 and γδ17 T cells (A) and different γδ T cell subsets (B) in pLNs of mid-aged mice under treatment with control isotype IgG2b or anti-IL-7 neutralising antibody. **(C)** Proportion of $V\gamma6^+$ T cells in the pLN γδ T cell pool. **(D)** Proliferation of γδ1 and γδ17 T cells within each γδ T cell subset in pLNs. **(E and F)** Proliferation of bulk $CD4^+$ T cells and $CD44^{hi}$ memory $CD4^+$ T cells (E), as well as bulk $CD8^+$ T cells and $CD44^{hi}$ memory $CD8^+$ T cells (F) in pLNs. Results shown are collected from an experiment with 9 mid-aged mice (5 for isotype control group and 4 for experimental group). Statistical significances for changes in expression levels were assessed by two-way ANOVA (A-C) or Mann-Whitney test (D-F). Error bars represent SD. *p<0.05; **p<0.01; ***p<0.001; ****p<0.0001

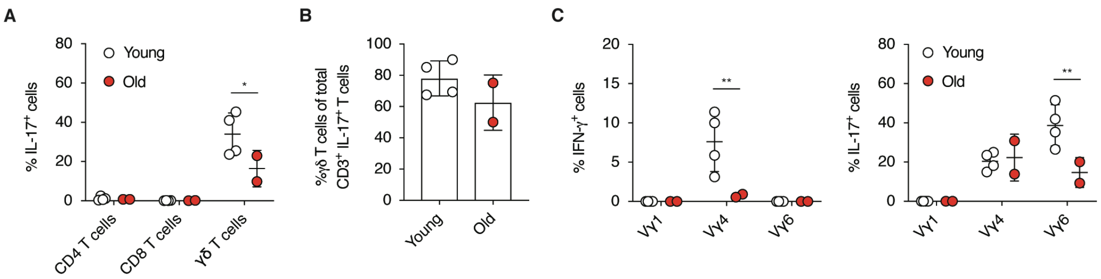

Appendix Figure S9. IL-17 production by tumour-infiltrating T cells. Total immune cells were extracted from 3LL-A9 tumours of young and old mice on day 14, and stimulated *ex vivo* with PMA and ionomycin for 4 hours in the presence of GolgiSTOP. **(A)** IL-17 production by CD4+ T cells, CD8+ T cells as well as γδ T cells. **(B)** Proportion of γδ T cells in total IL-17-producing CD3+ T lymphocytes. **(C)** IL-17 and IFN-γ production by different γδ T cell subsets from the tumour of young and old mice. Results shown are obtained from an experiment with 4 young and 2 old mice. Statistical significances for differences were assessed by two-way ANOVA (A and C) or Mann-Whitney test (B). Error bars represent SD. *p<0.05; **p<0.01

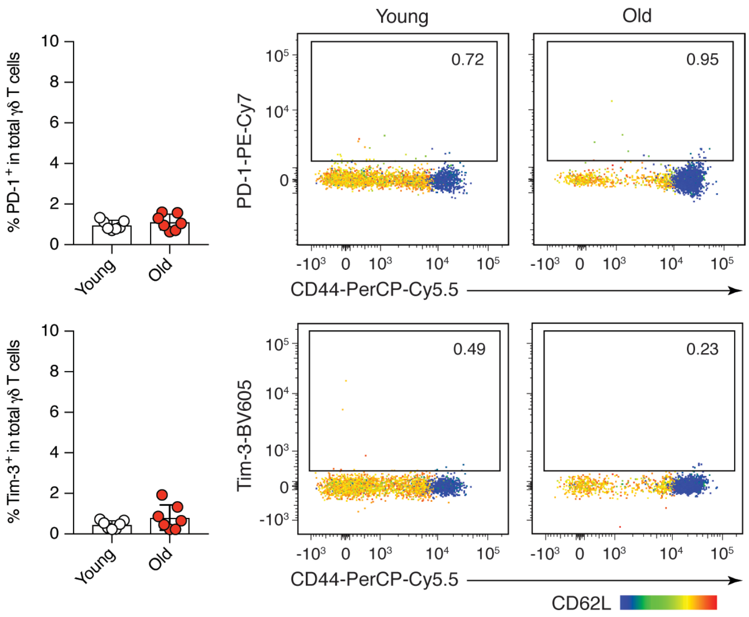

Appendix Figure S10. Expression of PD-1 and Tim-3 by γδ T cells in the pLNs of young and old mice under homeostatic condition. PD-1 (top row) and Tim-3 (bottom row) expression by γδ T cells in the pLNs of young and old mice was analysed by flow cytometry. Results shown are from 3 experiments with 7 young and 7 old mice. Statistical significance for changes were assessed by Mann-Whitney test. Error bars represent SD.

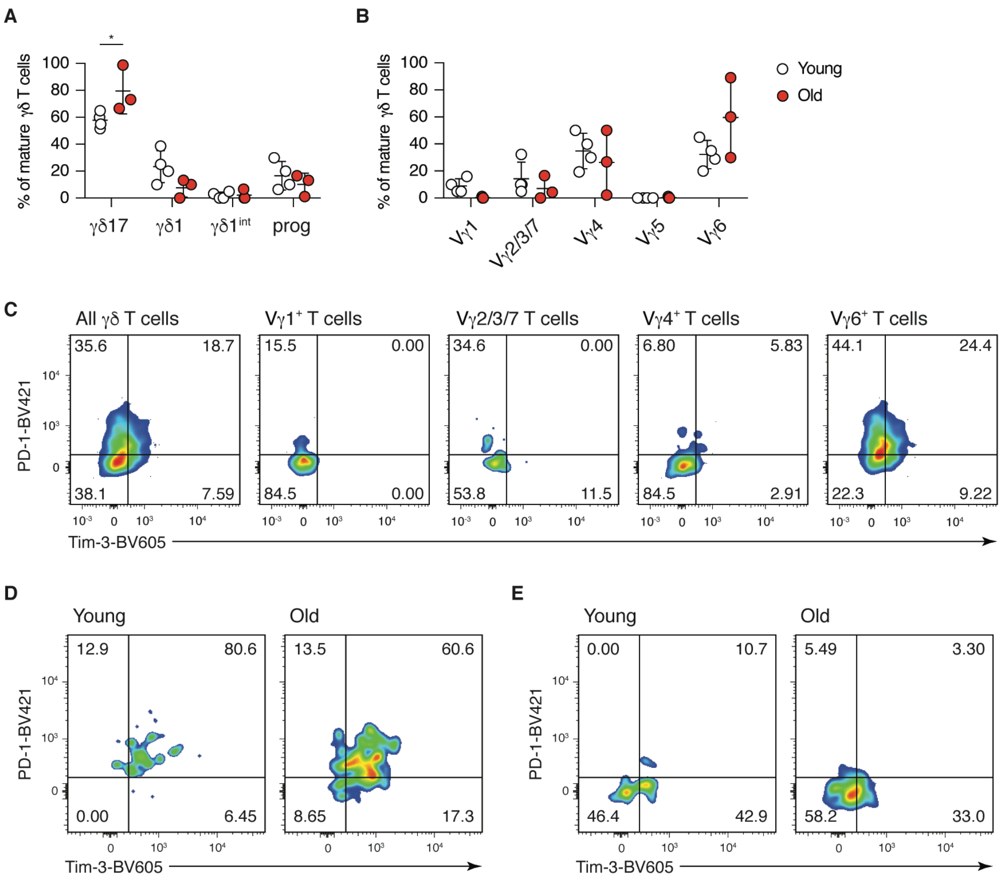

Appendix Figure S11. Activation of γδ T cell subsets in LL2 tumours and tumour-draining LNs of young and old mice. Young and old mice were injected subcutaneously with 3x10^6 LL2 Lewis lung carcinoma cells. Tumours and tumour-draining LNs were harvested at day 11 or day 14 for FACS analysis. **(A and B)** γδ1 and γδ17 lineage commitment (A) and proportion (B) of γδ T cells in the tumours of young and old mice on day 14. **(C)** Activation and exhaustion status of each γδ T cell subset in the tumour of young mice was characterised by PD-1 and Tim-3 expression on day 11. **(D and E)** Activation and exhaustion status of Vγ6+ T cells in tumour (D) and tumour-draining LN (E) of young and old mice at day 14. FACS files acquired from each individual mouse were concatenated for the analysis and the results are shown as representative dot plots. Results shown in (A, B, D and E) were obtained from an experiment with 4 young and 3 old mice. Results shown in (C) are obtained from an experiment with 5 young mice. Statistical significances for differences were assessed by two-way ANOVA (A and B). Error bars represent SD. *p<0.05

Appendix Table S1. Antibodies used in this study

Antigen	Clone	Dilution	Manufacturer	Identifier	Conjugate
CD3ε	145-2C11	1:50	BD	Cat #563565	BUV395
CD4	RM4-5	1:400	Biolegend	Cat #100516	APC
CD4	RM4-5	1:50	Biolegend	Cat #100559	BV510
CD4	RM4-5	1:200	Biolegend	Cat #100548	BV605
CD4	RM4-5	1:200	Biolegend	Cat #100546	BV650
CD4	GK1.5	1:200	BD	Cat #564667	BUV496
CD8α	53-6.7	1:50	Biolegend	Cat #100752	BV510
CD8α	53-6.7	1:200	Biolegend	Cat #100744	BV605
CD8α	53-6.7	1:200	Biolegend	Cat #100742	BV650
CD8α	53-6.7	1:200	Biolegend	Cat #100748	BV711
CD8α	53-6.7	1:200	Biolegend	Cat #100750	BV785
CD8	53-6.7	1:200	BD	Cat #564297	BUV737
CD11b	M1/70	1:100	Biolegend	Cat #101237	BV605
CD19	6D5	1:50	Biolegend	Cat #115546	BV510
CD24	M1/69	1:200	Biolegend	Cat #101808	PE
CD24	M1/69	1:200	Biolegend	Cat #101826	BV421
CD24	M1/69	1:200	BD	Cat #563545	BV650
CD27	LG.7F9	1:100	eBioscience	Cat #25-0271-82	PE-Cy7
CD44	IM7	1:200	Biolegend	Cat #103047	BV605
CD44	IM7	1:200	Biolegend	Cat #103059	BV785
CD45	30-F11	1:300	Biolegend	Cat #103133	BV421
CD45RB	C363-16A	1:200	Biolegend	Cat #103314	PerCP-Cy5.5
CD62L	MEL-14	1:400	Biolegend	Cat #104441	BV510
CD127	A7R34	1:50	Biolegend	Cat #135035	BV711
IL-23R	12B2B64	1:100	Biolegend	Cat #150904	PE
CCR6	140706	1:100	BD	Cat #564736	BV421
PD-1	RMP1-30	1:50	Biolegend	Cat #109121	BV421
PD-1	RMP1-30	1:50	Biolegend	Cat #109110	PE-Cy7
Tim-3	RMT3-23	1:50	Biolegend	Cat #119721	BV605
F4/80	BM8	1:50	Biolegend	Cat #123108	FITC
Ly6C	HK1.4	1:400	Biolegend	Cat #	PE
Ly6G	1A8	1:200	Biolegend	Cat #127614	APC
IFN-γ	XMG1.2	1:50	Biolegend	Cat #505836	BV711
IL-17A	TC11-18H10.1	1:50	Biolegend	Cat #506926	BV421
TCRβ	H57-597	1:50	Biolegend	Cat #109233	BV510
TCRβ	H57-597	1:50	Biolegend	Cat #109243	BV711
TCRβ	H57-597	1:200	Biolegend	Cat #109204	biotin
TCRγδ	UC7-13D5	1:100	Biolegend	Cat #107507	PE
TCRδ	GL3	1:100	Biolegend	Cat #118128	AF488
TCRδ	GL3	1:50	Biolegend	Cat #118116	APC
TCRδ	GL3	1:50	Biolegend	Cat #118131	BV510
TCRδ	GL3	1:100	Biolegend	Cat #118129	BV605

Appendix Table S1. Antibodies used in this study (continued)

Antigen	Clone	Dilution	Manufacturer	Identifier	Conjugate
TCR Vγ1	2.11	1:100	Biolegend	Cat #141108	APC
TCR Vγ4	UC3-10A6	1:50	Biolegend	Cat #137704	FITC
TCR Vγ4	UC3-10A6	1:100	Biolegend	Cat #137704	PE
TCR Vγ5	536	1:100	Biolegend	Cat #137506	APC
TCR Vγ5	536	1:100	Biolegend	Cat #137504	PE
TCR Vγ5/ Vγ6	17D1*	30 μl/sample	Prof. Adrian Hayday	-	-
rat IgM	RM-7B4	1:100	eBioscience	Cat #12-4342-82	PE
CD16/32 (TruStain fcX)	93	1:100	Biolegend	Cat #101320	-
IL-7	M25	4 mg/kg	BioXCell	Cat #BE0048	-
IgG2b	MPC-11	4 mg/kg	BioXCell	Cat #BE0086	-
TCRγδ	UC7-13D5	100 μg/mouse	BioXCell	Cat #BE0070	-
Armenian hamster IgG	polyclonal	100 μg/mouse	BioXCell	Cat #BE0091	-

*hybridoma supernatant

Appendix Table S2. Gene nomenclatures of TCRδ variable segments*

Subgroup	*IMGT gene name [1-3]*	*Arden et al. [4]*	*Previously designated Vδ*	*Designated Vδ in this study*
**TRDV1**	TRDV1	DV102S1	**Vδ2 [5]**	**Vδ2**
**TRDV2§**	TRDV2-1	-	-	**Vδ4**
	TRDV2-2	DV104S1	**Vδ4 [6]**	**Vδ4**
**TRDV3**	TRDV3	-	***pseudogene***	**-**
**TRDV4**	TRDV4	DV101S1	**Vδ1 [5]**	**Vδ1**
**TRDV5**	TRDV5	DV105S1	**Vδ5 [6]**	**Vδ5**
**TRDV6§**	TRAV15-1/DV6-1	ADV7S2	**Vδ6 [5] and Vδ6.1 [7]**	**Vδ6**
	TRAV15-2/DV6-2	DV7S4	**Vδ6 [6]**	**Vδ6**
	TRAV15D-1/DV6D-1	ADV7S1/DV7S6	**Vδ6.3 [8] / Vδ6.2 [7]**	**Vδ6**
	TRAV15D-2/DV6D-2	DV7S5	**Vδ6 [6]**	**Vδ6**
**TRDV7**	TRAV13-4/DV7	DV10S7	**Vδ7 [9]**	**Vδ7**
**TRDV8**	TRAV14D-3/DV8	DV2S8	**Vδ8 [8]**	**Vδ8**
**TRDV9**	TRAV6-7/DV9	DV4S8	**Not designated [10]**	**-**
**TRDV10**	TRAV4-4/DV10	ADV11S5	**Vδ [11]**	**-**
**TRDV11**	TRAV16D/DV11	AV17S1/ADV17S2	**Vδ9 [12]**	**Vδ9**
**TRDV12**	TRAV21/DV12	DV6S2	**Vδ3 [6]**	**Vδ3**

*This table is modified from Arden et al., 1995 [4], and Bosc and Lefranc, 2003 [3].
§For clarity, the gene segments belong to the same subgroup are merged as one in this study.

Reference

1. Bosc, N., V. Contet, and M.P. Lefranc, *The mouse (Mus musculus) T cell receptor delta variable (TRDV), diversity (TRDD) and joining (TRDJ) genes.* Exp Clin Immunogenet, 2001. **18**(1): p. 51-8.
2. Glusman, G., et al., *Comparative genomics of the human and mouse T cell receptor loci.* Immunity, 2001. **15**(3): p. 337-49.
3. Bosc, N. and M.P. Lefranc, *The mouse (Mus musculus) T cell receptor alpha (TRA) and delta (TRD) variable genes.* Dev Comp Immunol, 2003. **27**(6-7): p. 465-97.
4. Arden, B., et al., *Mouse T-cell receptor variable gene segment families.* Immunogenetics, 1995. **42**(6): p. 501-30.
5. Chien, Y.H., et al., *T-cell receptor delta gene rearrangements in early thymocytes.* Nature, 1987. **330**(6150): p. 722-7.
6. Elliott, J.F., et al., *The adult T-cell receptor delta-chain is diverse and distinct from that of fetal thymocytes.* Nature, 1988. **331**(6157): p. 627-31.
7. McConnell, T.J., et al., *Delta-chains of dendritic epidermal T cell receptors are diverse but pair with gamma-chains in a restricted manner.* J Immunol, 1989. **142**(8): p. 2924-31.
8. Happ, M.P., et al., *Limited receptor repertoire in a mycobacteria-reactive subset of gamma delta T lymphocytes.* Nature, 1989. **342**(6250): p. 696-8.
9. Takagaki, Y., et al., *T cell receptor-gamma and -delta genes preferentially utilized by adult thymocytes for the surface expression.* J Immunol, 1989. **142**(6): p. 2112-21.
10. Korman, A.J., et al., *Predominant variable region gene usage by gamma/delta T cell receptor-bearing cells in the adult thymus.* J Exp Med, 1988. **168**(3): p. 1021-40.
11. Bluestone, J.A., et al., *Structure and specificity of T cell receptor gamma/delta on major histocompatibility complex antigen-specific CD3+, CD4-, CD8- T lymphocytes.* J Exp Med, 1988. **168**(5): p. 1899-916.
12. Takeshita, S., M. Toda, and H. Yamagishi, *Excision products of the T cell receptor gene support a progressive rearrangement model of the alpha/delta locus.* EMBO J, 1989. **8**(11): p. 3261-70.
