# Supplementary material for: IL‐7‐dependent compositional changes within the γδ T cell pool in lymph nodes during ageing lead to an unbalanced anti‐tumour response
Source: EMBO Rep. 2019 Jul 8;20(8):e47379. doi: 10.15252/embr.201847379 (PMC6680116; doi:10.15252/embr.201847379)
Supplement: Supplementary file 2 — Expanded View Figures PDF [file EMBR-20-e47379-s002.pdf]

Expanded View Figures

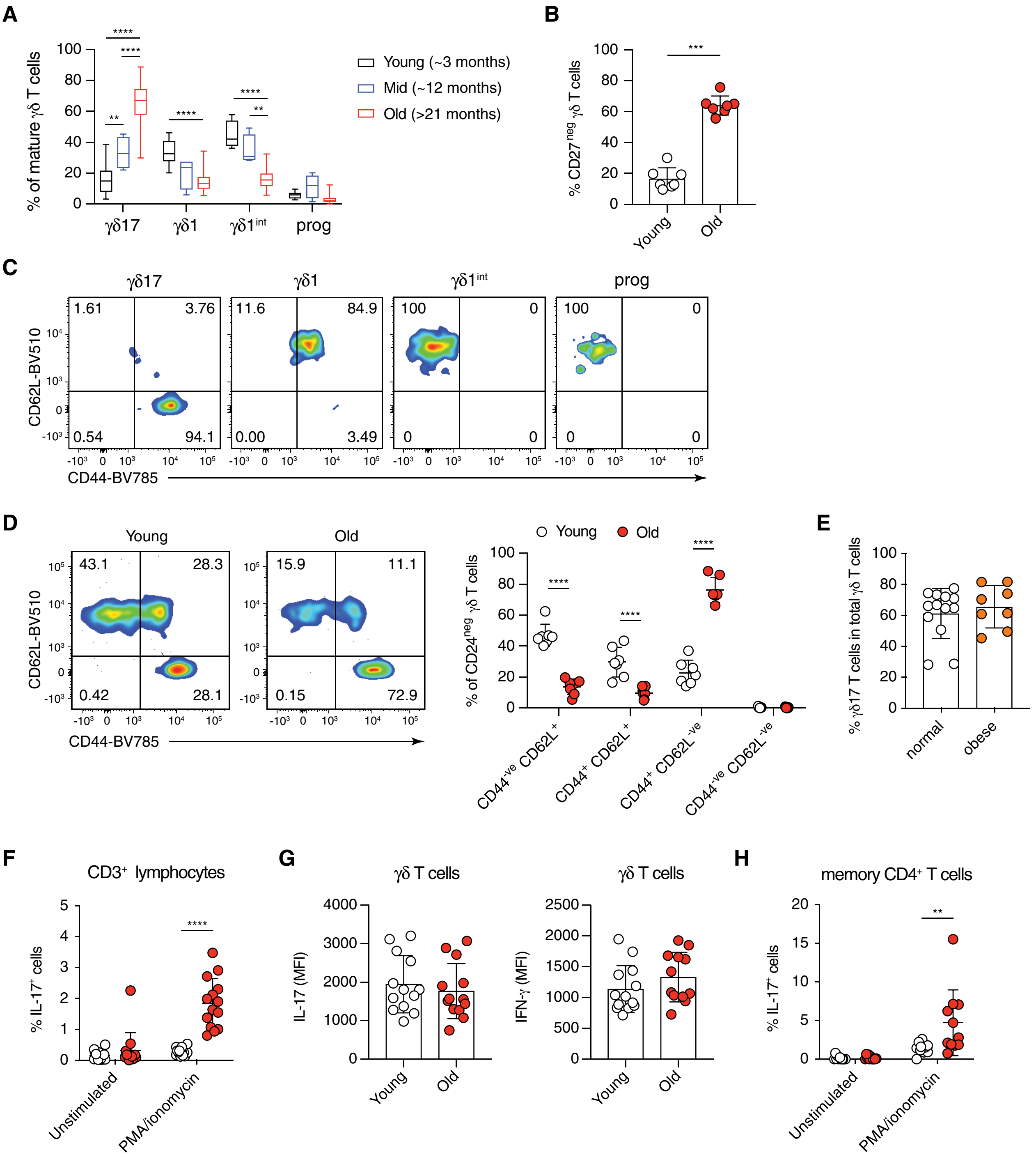

Figure EV1.

 © 2019 The Authors

Figure EV1. Lineage polarisation, phenotype and function of γδ T cells in the pLNs of young and old mice.

A The expression of CD45RB and CD44 segregates mature ($CD24^{neg}$) γδ T cells in LNs of young (3 months, *n* = 17), mid-age (12 months, *n* = 4) and old (> 21 months, *n* = 16) mice into γδ17-committed ($CD45RB^{neg}$ $CD44^{+}$), γδ1-committed ($CD45RB^{+}$ $CD44^{+}$), γδ1-intermediate ($CD45RB^{+}$ $CD44^{neg}$) and progenitor ($CD45RB^{neg}$ $CD44^{neg}$) γδ T-cell subsets.

B Percentage of γδ17 T cells, as characterised by the lack of CD27 expression, from total γδ T cells in old and young pLNs. Results shown are from three independent experiments with seven young and seven old mice.

C CD44 and CD62L expression profile of γδ17-committed ($CD45RB^{neg}$ $CD44^{hi}$), γδ1-committed ($CD45RB^{+}$ $CD44^{+}$), γδ1-intermediate ($CD45RB^{+}$ $CD44^{neg}$) and progenitor ($CD45RB^{neg}$ $CD44^{neg}$) γδ T cells from pLNs of young mice.

D Representative FACS plots and analysis of the memory/activation status of γδ T cells in the pLNs of young and old mice. Results shown are collected from three independent experiments using seven young and seven old mice.

E Effect of obesity on γδ17 bias in the pLNs of old mice. Aged mice with obesity were visually identified across three ageing cohorts in the same animal facility. The proportion of γδ17 T cells in the pLN γδ T-cell pool of normal and obese old mice was compared. Results shown are collected from 11 independent experiments with 21 old mice (13 normal and eight obese).

F–H Cytokine production by total $CD3^{+}$ T cells (F), γδ T cells (G) and $CD44^{hi}$ memory $CD4^{+}$ T cells (H) in the pLNs of young and old mice after *ex vivo* stimulation with PMA and ionomycin for 4 h in the presence of GolgiSTOP. Results shown in (G) are collected from six independent experiments with 16 young and 15 old mice. Results shown in (F) and (H) are collected from five experiments with 13 young and 12 old mice.

Data information: Statistical significance for changes was assessed using Mann–Whitney test (B, E and G) or two-way ANOVA (A, D, F and H). Error bars represent SD. In the box plots (A), lower and upper hinges indicate the first and third quartile, and the horizontal line within the box indicates the median. Upper whiskers extend from Q3 to the maximum and lower whiskers from Q1 to the minimum value. $^{**}P < 0.01$; $^{***}P < 0.001$; $^{****}P < 0.0001$.

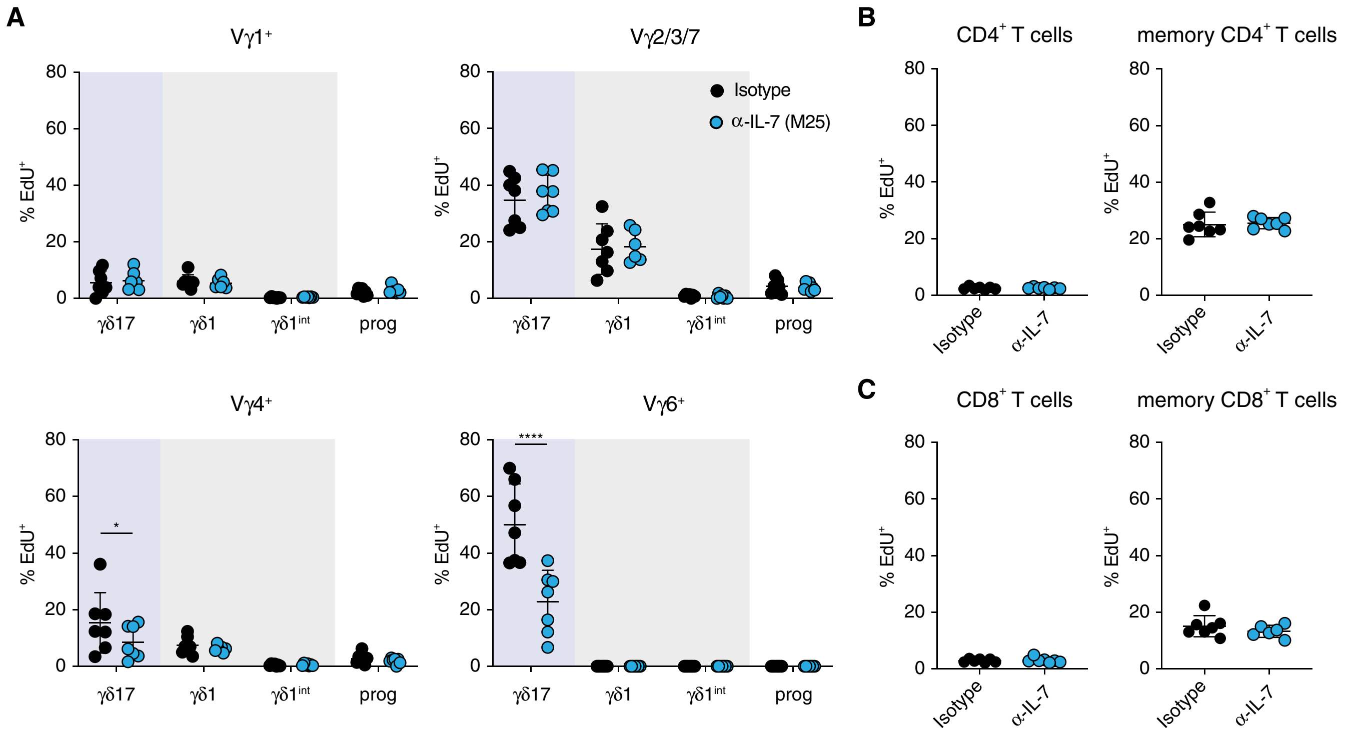

Figure EV2. IL-7-dependent proliferation of γδ17 T cells in pLNs of young mice.

Mice were treated with 4 mg/kg IgG2b or anti-IL7 antibody by *i.p.* injection followed by administration of EdU by *i.p.* injection and supply in drinking water (as shown in Fig 5I). The level of proliferation was assayed by the level of EdU incorporation over a period of 3 days.

A The proliferation of γδ1 and γδ17 T cells within each γδ T-cell subsets under treatment with control isotype IgG2b or with anti-IL-7 neutralising antibody.

B, C The proliferation of bulk $CD4^{+}$ T cells and $CD44^{hi}$ memory $CD4^{+}$ T cells (B), as well as bulk $CD8^{+}$ T cells and $CD44^{hi}$ memory $CD8^{+}$ T cells (C), under treatment with control isotype IgG2b or with anti-IL-7 neutralising antibody.

Data information: Results shown are collected from two independent experiments with 14 young mice (seven each for control and experimental groups). Statistical significances for changes in expression levels were assessed by two-way ANOVA (A) or Mann–Whitney test (B and C). Error bars represent SD. $^{*}P < 0.05$; $^{****}P < 0.0001$.

© 2019 The Authors

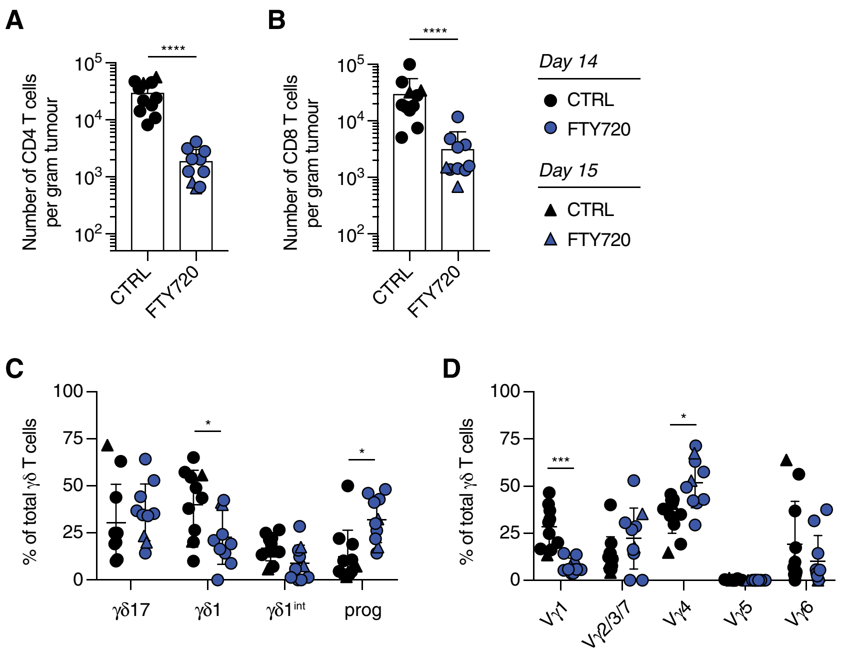

Figure EV3. Egress of γδ and αβ T cells from pLNs into the tumour.

Young mice were injected every other day by *i.p.* with FTY720 at a dose of 1 mg/kg or with vehicle control containing 2.5% ethanol and 2% β-cyclodextrin from day −5 to day 13. 3×10^6 3LL-A9 cells were given to control and FTY720-treated mice by subcutaneous injection on day 0. Tumours were harvested at day 14 or 15 for FACS analysis.

A, B Number of $CD4^+$ (A) and $CD8^+$ (B) T cells in the tumour of control and FTY720-treated mice.

C γδ T-cell lineages observed in the tumour of control and FTY720-treated mice.

D Composition of γδ T cells subsets in the tumour of control and FTY720-treated mice.

Data information: Results shown are obtained from two independent experiments with 11 control and 10 FTY720-treated mice. Statistical significances for the difference in cell densities and cell proportions were assessed by Mann–Whitney test (A and B) or two-way ANOVA (C and D). Error bars represent SD. *$P < 0.05$; ***$P < 0.001$; ****$P < 0.0001$.

© 2019 The Authors

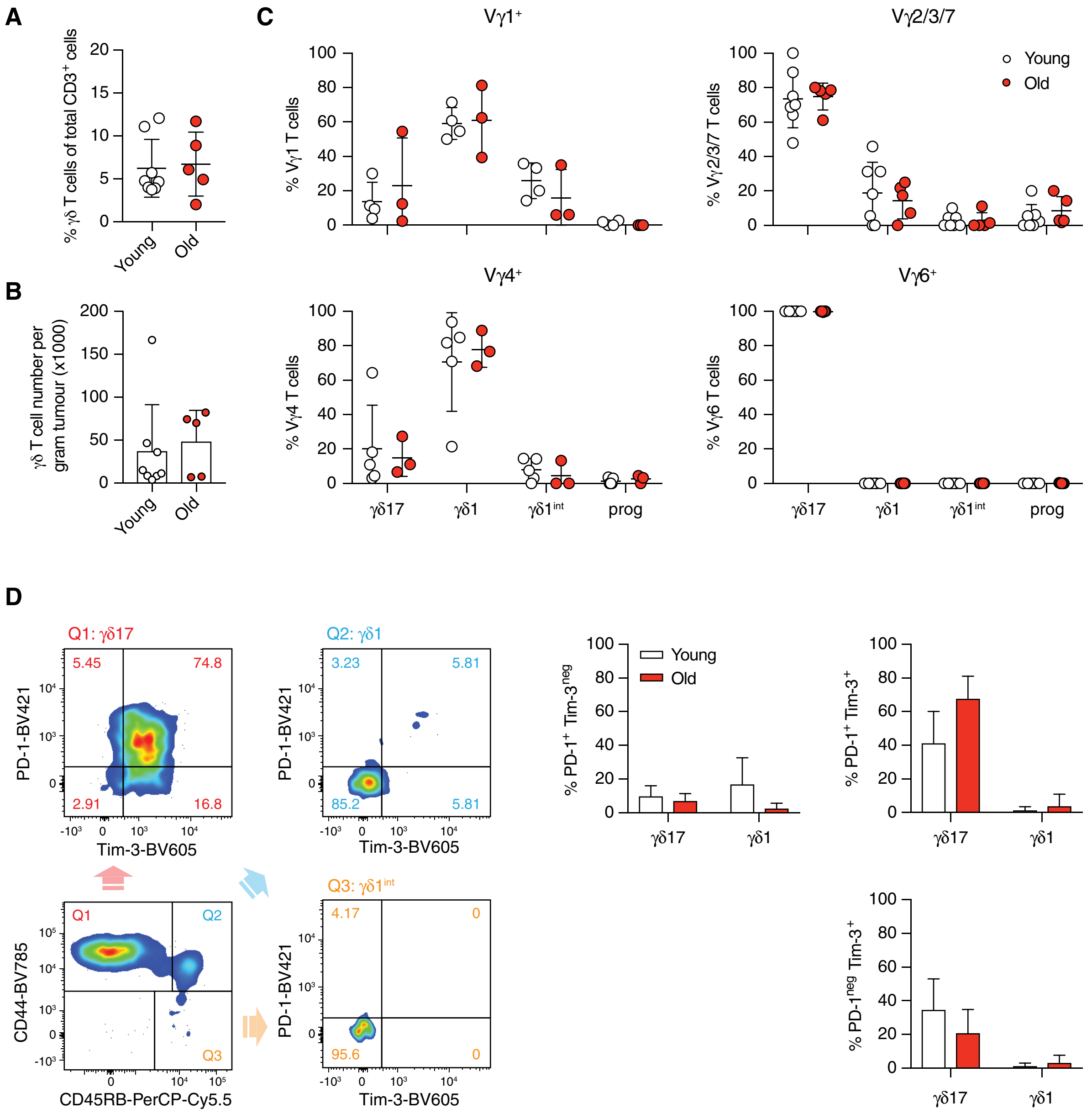

Figure EV4. Activation and exhaustion status of different γδ T-cell subsets in the tumour of young and old mice.

3LL-A9 Lewis lung carcinoma cells were injected subcutaneously into young and old mice, and tumours were analysed 14 days after injection.

A Percentage of γδ T cells in total tumour-infiltrating $CD3^+$ T lymphocytes.

B Density of γδ T cells in the tumours of young and of mice.

C γδ1 and γδ17 lineage commitment of tumour-infiltrating $V\gamma1^+$, Vγ2/3/7, $V\gamma4^+$ and $V\gamma6^+$ T cells.

D Activation of γδ1 and γδ17 T-cell subsets in the tumour as determined by their PD-1 and Tim-3 expression profile. Representative FACS plots (left) show the analysis with concatenated FACS data acquired for each individual young mouse. Results shown (right) are obtained from two independent experiments with eight young and five old mice. Cell populations with a total cell number < 10 were excluded from the analysis.

Data information: Statistical significances for differences were assessed by Mann–Whitney test (A and B) or two-way ANOVA (C and D). Error bars represent SD.

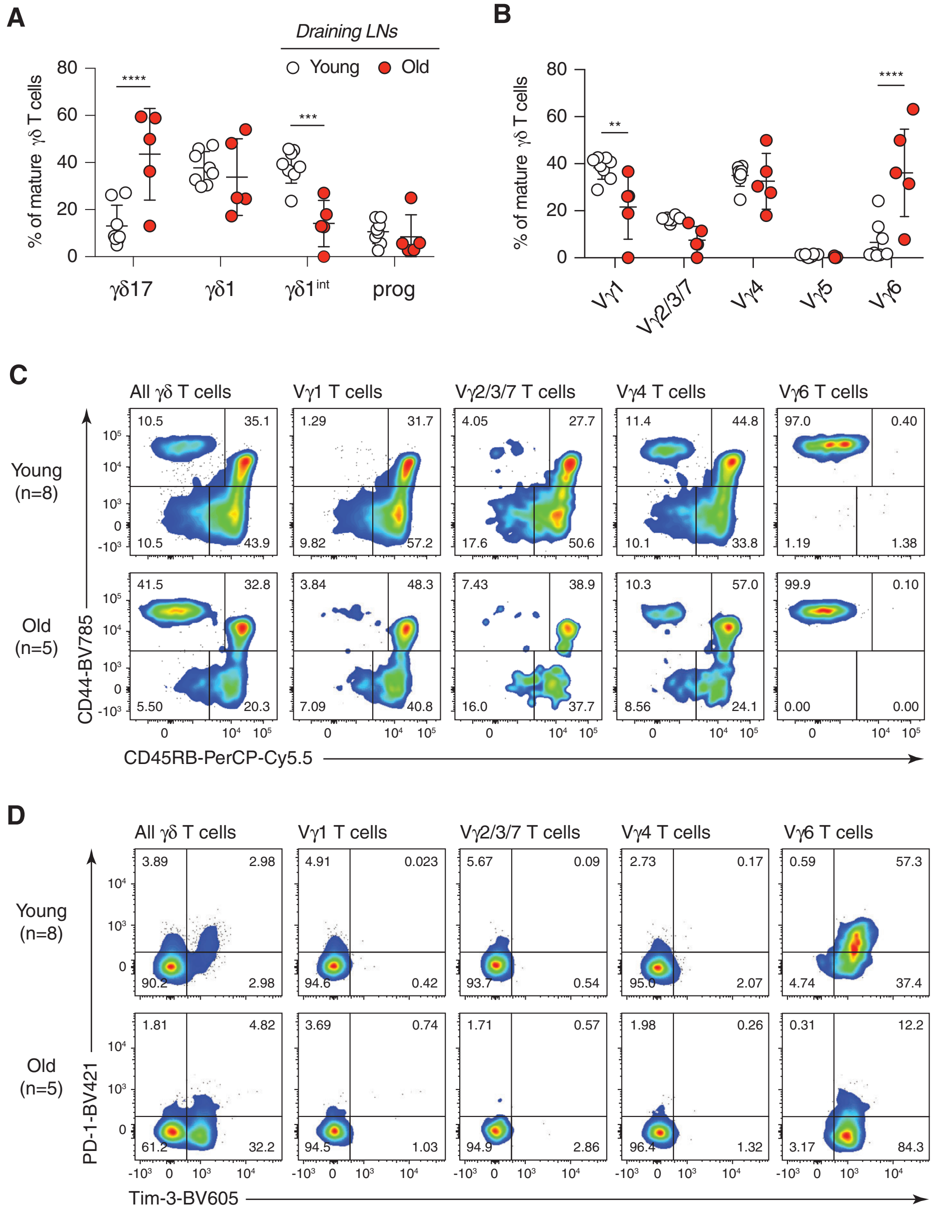

Figure EV5. Activation and exhaustion status of different γδ T-cell subsets in the draining LN of tumour-bearing young and old mice.

A γδ1 and γδ17 lineage commitment of γδ T cells in the tumour-draining LN of young and old mice.

B Proportion of γδ T-cell subsets in the tumour-draining LN of young and old mice.

C γδ1 and γδ17 lineage commitment of each γδ T-cell subset in the tumour-draining LN was characterised by CD44 and CD45RB expression.

D Activation and exhaustion status of each γδ T-cell subset in the tumour-draining LN was characterised by PD-1 and Tim-3 expression.

Data information: FACS files acquired for each individual mouse were concatenated for the analysis, and the results are shown as representative dot plots. Results are obtained from two independent experiments with eight young and five old mice. Cell populations with the total cell number < 10 were excluded from analyses. Statistical significances for differences were assessed by two-way ANOVA (A and B). Error bars represent SD. $^{**}P < 0.01$; $^{***}P < 0.001$; $^{****}P < 0.0001$.

© 2019 The Authors
